# A cluster randomized controlled trial comparing three methods of disseminating practice guidelines for children with croup [ISRCTN73394937]

**DOI:** 10.1186/1748-5908-1-10

**Published:** 2006-04-28

**Authors:** David W Johnson, William Craig, Rollin Brant, Craig Mitton, Larry Svenson, Terry P Klassen

**Affiliations:** 1 Department of Pediatrics, Faculty of Medicine, University of Calgary, Calgary Alberta, Canada; 2 Department of Pediatrics, Faculty of Medicine, University of Alberta, Edmonton, Alberta, Canada; 3 Department of Statistics, University of British Columbia, Vancouver, British Columbia, Canada; 4 Faculty of Health and Social Development, University of British Columbia- Okanagan, Kelowna, British Columbia, Canada; 5 Health Surveillance, Alberta Health and Wellness, Edmonton, Alberta, Canada

## Abstract

**Background:**

The optimal management of croup – a common respiratory illness in young children – is well established. In particular, treatment with corticosteroids has been shown to significantly reduce the rate and duration of intubation, hospitalization, and return to care for on-going croup symptoms. Furthermore treatment with a single dose of corticosteroids does not appear to result in any significant adverse outcomes, and yields overall cost-savings for both families and the health care system.

However, as has been shown with many other diseases, there is a significant gap between what we know and what we do. The overall aim of this study is to identify, from a societal perspective, the costs and associated benefits of three strategies for implementing a practice guideline that addresses the management of croup.

**Methods/designs:**

We propose to use a matched pair cluster trial in 24 Alberta hospitals randomized into three intervention groups. We will use mixed methods to assess outcomes including linkage and analysis of administrative databases obtained from Alberta Health and Wellness, retrospective medical chart audit, and prospective telephone surveys of the parents of children diagnosed to have croup. The intervention strategies to be compared will be mailing of printed educational materials (low intensity intervention), mailing plus a combination of interactive educational meetings, educational outreach visits, and reminders (intermediate intensity intervention), and a combination of mailing, interactive sessions, outreach visits, reminders plus identification of local opinion leaders and establishment of local consensus processes (high intensity intervention). The primary objective is to determine which of the three intervention strategies are most effective at lowering the rate of hospital days per 1,000 disease episodes. Secondary objectives are to determine which of the three dissemination strategies are most effective at increasing the use of therapies of known benefit. An economic analysis will be conducted to determine which of the three intervention strategies will most effectively reduce total societal costs including all health care costs, costs borne by the family, and costs stemming from the strategies for disseminating guidelines.

## Background

### Introduction

Compared with biomedical research, few resources have been devoted to translating knowledge into practice. However health care researchers are increasingly aware of the persistent and substantial gap between what we know and what we do. One of the most common strategies advocated for closing this gap is the development of practice guidelines. It is now widely accepted, however, that simple dissemination whether by publication in medical journals or direct mailings to physicians of guidelines, does not result in a significant change in practice.

More than 40 systematic reviews have been published which examine a range of different strategies for implementing guidelines. An overview of this literature has been published by the Cochrane Effective Practice and Organisation of Care (EPOC) Group.[1,2] This review concludes that many of the primary studies focusing on implementation strategies have weak designs, methodological flaws, and that virtually none have included economic evaluations. The authors call for randomized trials with head-to-head, multi-arm comparisons of different levels of interventions, that use an appropriate analysis, based on clusters, and include a comprehensive cost-effective analysis.

We have designed a study that addresses each of the points outlined by the EPOC Group's members. Furthermore we believe – for several reasons – that practice guidelines addressing the management of croup will provide a good test case for analyzing our proposed intervention.

Croup is second only to asthma as a cause of respiratory emergencies in young children, and accounts for 5% of all emergent admissions in this population. In the last 15 years, as a result of the publication of a number of therapeutic trials and systematic reviews, the scientific basis for croup treatment has been clarified. In particular, optimally timed treatment with steroids substantially reduces the frequency and duration of both hospitalization and airway intubation. [3-5]

Published literature and pilot data collected for this proposed trial show that many children with croup do not receive optimal therapy, and that there is a substantial variation in hospitalization rates.[6,7] Since hospitalization likely accounts for most health care spending on this disease, improvement in prescribing practices and standardization of indications for hospital admission could both improve outcomes and substantially reduce health care expenditures for children with croup.

#### Strategies for disseminating and implementing clinical practice guidelines

##### The "Gap"

On-going societal investment in basic and clinical research is ultimately only meaningful if this acquired knowledge is translated into better care for patients. Unfortunately, in health care, there is a substantial gap between what is known and what is actually done for patients. Though this has been recognized for some time, this gap has not disappeared. Some well-documented examples include the use of photocoagulation for diabetic retinopathy,[8] management of hypertension,[8] prophylaxis for patients at high risk for venous thrombosis and pulmonary embolism,[8] and, most recently, thrombolytic therapy for patients with myocardial infarction. [8-10]

##### Approaches to knowledge translation

Many different broad approaches to knowledge translation have been advocated including development and dissemination of clinical practice guidelines, continuing medical education, continuous quality improvement, and more recently, computerized decision support systems.[11,12]

##### What constitutes a good practice guideline?

Guidelines are formally defined as "systematically developed statements to assist practitioners' and patients' decisions about appropriate care for specified clinical outcomes".[13] A number of organizations have developed policies and standards for how guidelines should be developed and evaluated. [12-17] It is desirable for guidelines to be valid (i.e. if followed, guidelines should yield the health care gains and costs predicted); reproducible (i.e. if given the same evidence and methods, two committees should develop comparable guidelines); and reliable (i.e. in the same clinical circumstances, clinicians should interpret the guidelines in the same way).[9] Guidelines are most likely to be valid if developed by national organizations with representatives from all key disciplines, and if formally linked to meta-analysis.[9]

##### Guideline development is expensive and often falls short

Development of guidelines can be very expensive, especially those developed by national agencies which can range up to US $1 million.[17] Furthermore, there are more than 20,000 guidelines registered in the U.S. National Guideline Clearinghouse, and more than 2,000 guidelines on the Canadian Medical Association Website.[18] Consequently the total amount of health resource dollars now devoted to guideline development is considerable. For example, the United Kingdom spends an estimated 1.5% of the annual health budget on the development of national guidelines.

A critical review of practice guidelines found that, of the guidelines published in a selection of journals in the last decade, none meet all methodological standards, and few met more than 70% of standards.[19] The methodological area in which guidelines needed the greatest improvement was the identification, evaluation, and synthesis of scientific evidence.

##### Dissemination alone doesn't work

It is now widely accepted that no matter how well-developed practice guidelines are, simple dissemination does not result in a significant change in practice.[1,2] A number of different strategies to implement guidelines have been published. Cochrane Effective Practice And Organisation Of Care Group  has systematically reviewed the published literature, developed a taxonomy of interventions, and developed methodological criteria for assessing the quality of the evaluation of these interventions.

##### Implementation strategies: what works and what doesn't work

Based on published reviews by the Cochrane EPOC Group and others, we can broadly categorize the different implementation strategies into three groups as showing consistent, variable, or little or no effectiveness.[1,2] Those interventions that consistently have shown effectiveness include interactive educational meetings, educational outreach visits, reminders (either manual or computerized), and multifaceted interventions (defined as a minimum of two combined interventions). Interventions that have shown a range of effectiveness include audit and feedback, the use of local opinion leaders, local consensus processes, and patient-mediated interventions. Interventions that have consistently shown little or no effect are didactic educational meetings (lecture-format) and educational materials (distribution of recommendations for clinical care, including practice guidelines, audiovisual materials, and electronic publications).

### Management of children with croup

#### Clinical presentation and infectious etiology

Croup (acute laryngotracheo-bronchitis) is a common respiratory tract illness in young children. Parainfluenza virus is, by far, the most common cause of croup. Other important causes include influenza, adeno, and respiratory syncytial viruses.[20,21] The illness is characterized by a barking cough, hoarseness, inspiratory stridor, and often severe respiratory distress that can occur suddenly in the middle of the night. This can be frightening for parents. The barking cough is distinct for croup. It and the accompanying inspiratory stridor are easily recognizable by clinicians and, once educated, by parents.

#### Epidemiology & burden of the disease

It most commonly affects children between the ages of 6 months to 3 years.[21] The disease peaks biannually, with the largest peak between September and December and a second smaller peak between January and March.[22] Croup accounts for a significant proportion of paediatric emergency department (ED) visits and hospitalizations. The proportion of children evaluated as outpatients who are hospitalized range from 1–6% of those seen in private physician offices, to 7–31% of those evaluated in an ED.[21,23-25] Hospitalizations of children with croup are typically short, with the median duration being 48 hours. The potential for respiratory failure and death is what drives physicians to consider hospitalization. However, endotracheal intubation is uncommon (0.4–1.4% of hospitalized children), and death is exceptionally rare (0.5% of intubated children).[26]

#### Indications for hospital admission

Few studies have examined which children are at risk for respiratory failure. A retrospective cohort study of 527 hospitalized children suggested that the presence or absence of persistent sternal and chest wall indrawing may be an important clinical factor in determining risk.[27] A small prospective study suggests that the institution of a clinical pathway for croup in an ED – using explicit criteria for when it is safe to discharge children home – can significantly reduce admission rates and length of stay without any significant adverse events.[28]

#### Variability in hospitalization rates

Using the technique of small area analysis, To and Wennberg have reported a five and 17-fold difference, respectively, in the rate of hospitalization per capita among communities. Wennberg noted that the rate for croup and several other common pediatric problems, relative to adult medical conditions, was significantly more variable.[6,7]

#### BaselineUtilisation data from Alberta

Utilizing provincial administrative data, we have established health care utilization rates including the a) number of disease episodes, b) number of physician visits per disease episode, and c) number of hospital admissions and hospitals days per disease episode, for children with croup in Alberta for 6 years. Among the 107 Alberta hospitals, we found up to an 18 fold range in hospital admissions rates, ranging from16 to 287/1000 disease episodes. In general, those hospitals that evaluated larger numbers of children with croup had lower utilization rates. For example, the six largest volume hospitals diagnosed a total of 86,711 cases, and averaged 21 hospital admissions/1,000 disease episodes; and the 30 smallest volume hospitals diagnosed 13,750 cases and averaged 116 hospital admissions/1,000 disease episodes.

#### Costs associated with hospitalization

Though there are no comprehensive studies examining the health care costs of croup, one study suggests that the total 'costs' for hospitalization were almost three times that of the total costs for all ED visits.[23] In the case of bronchiolitis – which likely has a similar costing structure to croup – 62% of all health care expenditures for the disease are expended on the less than 1% of children who are admitted to hospital.[29] Our baseline provincial data for croup shows that the average number of MD visits per disease episode is only 1.15, and that 3.6% of children with croup are hospitalised. Given the relative greater costs associated with hospital admission versus MD visits, or other costs such as medications, it is likely that hospital admissions are the principal determinant of health expenditures. Therefore a substantial reduction in hospitalization rates should substantially reduce health care expenditures for the disease.

#### Overview of current therapy

Treatment for croup includes oxygen, mist, epinephrine, corticosteroids, and heliox.[30,31] Though mist has been used for a long time, there is little evidence for or against its benefit.[32] The evidence includes small randomised and non-randomized trials, a study that used an animal model of uncertain applicability, and, recently, a well-masked randomised trial published by one of us (TPK). [33-36] None of these studies found mist to be beneficial.

Epinephrine is clearly effective in the short-term, based on several randomised trials using clinical scores, and non-controlled trials using a range of 'objective' measures to assess degree of respiratory distress.[34,37-40] In patients with severe respiratory obstruction, its use probably prevents or delays the need for endotracheal intubation.[40,41] Nebulized epinephrine is generally thought to be safe.[30,31,42] Since the effect of epinephrine, however, is short-lived and does not alter the natural history of the disease,[38] there appears to be no real benefit to its use in children with mild symptoms.

Heliox (helium mixed with oxygen) allows efficient laminar flow in narrower airways, and, at least in theory, can delay or even prevent endotracheal intubation. [43] Two small randomized trials in children with moderately severe croup have been published. One compared heliox to nebulized epinephrine and found the two interventions to be equivalent.[44] The other study compared heliox to standard oxygen therapy, and found a trend in favor of heliox that was not statistically significant.[45]

The clinical benefit of steroids is well documented. In addition to observations that steroids improve clinical scores, randomized trials have shown a reduction in the duration of endotracheal intubation,[46] the rate of intubation, [4] the amount of treatment required with epinephrine,[3,5] the duration of hospitalization,[47] the rate of hospitalization, [5,48] and the rate of return to the ED.[49] Steroid therapy for croup is generally thought to be safe. [31]

#### Unsubstantiated therapies: antibiotics, decongestants, & beta-agonists

Other types of therapies sometimes administered to patients with croup include antibiotics, decongestants, and nebulized β-agonists. No evidence exists for their benefit.

#### Variability in treatment practices

A recent study reported significant differences in the proportion of children treated with mist, epinephrine, and salbutamol based on whether physicians were pediatricians or not.[50] All physicians in these two urban hospitals, however, used corticosteroids in more than 90% of children with croup. In contrast, our data from Alberta shows that a much smaller proportion of children receive corticosteroids, (e.g. only 22% at the 12 smaller hospitals). In these same small hospitals, the same proportion of children receive antibiotics or salbutamol; treatments for which there is no evidence of efficacy.

#### Why are guidelines that address the management of croup a good choice for testing implementation strategies?

First, croup is an extremely common disease that is seen in small rural health hospitals as well as in tertiary centres. Second, a large body of published evidence clearly demonstrates that properly timed treatment with corticosteroids can significantly reduce health care utilization, without any significant adverse events. Furthermore, understanding what constitutes best therapy is not difficult to grasp. Third, it is a reasonable assumption that hospitalization is the primary determinant of health care expenditures for the disease. Fourth, there is a substantial variation in practice patterns and hospitalization rates across Alberta. Therefore effective implementation strategies should lower overall health care expenditures and increase societal benefits.

### Relevant systematic reviews

#### Strategies for implementing practice guidelines

Cochrane EPOC Group members have published two 'overviews' of systematic reviews of knowledge translation interventions.[1,2] In general, these systematic reviews found, that despite the relatively large number (~ 1,600) of published articles which address the topic, only a comparatively small number (~ 100) have a semi-rigorous design (randomised, quasi-randomised, controlled before and after studies).[51] Many of these primary studies are methodologically flawed in that they randomised subjects by clusters, but used standard statistical techniques for analyzing their results.[52] Only a comparatively small number of trials have systematically examined the impact of implementation strategies on both medical practice and clinical outcomes, and very few studies have addressed the cost effectiveness of clinical guidelines.[1]

The Cochrane EPOC Group has established a taxonomy for classifying different types of implementation strategies. Educational meetings (defined as participation of health care providers in conferences, lectures, or workshops) show a range of effectiveness.[1] Interactive workshops have shown moderate to moderately large effects, whereas didactic forums alone were ineffective.[1] Educational outreach visits, (defined as the use of a trained person who meets with providers in their practice settings to provide information with the intent of changing providers' performances), have shown small to moderate effects on behaviour, though the authors point out that the cost effectiveness is unclear. Reminders (defined as any intervention, manual or computerised, that prompts the health care provider to perform a clinical action) appear to be more effective than audit and feedback but the results have not been striking.[1] The identification of local opinion leaders (defined as health professionals nominated by their colleagues as 'educationally influential') has shown mixed results in studies published to date, and the authors of this Cochrane systematic review suggested that further research was required before this technique becomes widespread.[1,2,53] Probably the most consistent finding among systematic reviews is that multi-faceted interventions (> 2 interventions of any type) are more effective than single interventions.[1,2]

#### Management of children with croup

Two meta-analyses of randomized controlled trials examining the benefit of corticosteroids have been published. Kairys et al. published a meta-analysis in 1989 of 10 published randomized controlled trial's involving 1286 patients.[4] The analysis indicated that the use of steroids in children hospitalized with croup is associated with a significantly increased proportion of children showing clinical improvement at 12 and 24 hours following treatment, and a significantly reduced incidence of endotracheal intubation. More recently, we (TPK and DWJ) performed a meta-analysis of 24 randomized controlled trial's, and found that corticosteroid treatment was associated with an improvement in croup score at 6, 12, and 24 hours, a decrease in epinephrine treatments, a decrease in length of time spent in the emergency department, and a reduction in hospital stay by 16 hours.[3]) A Cochrane protocol has been submitted to examine humidified air inhalation for treating croup, but has not been published yet. No systematic reviews have been published or registered for either epinephrine or heliox.

### Aim and objectives

The overall aim of this study is to identify, from a societal perspective, the costs and associated benefits of three strategies (of low, intermediate and high intensity respectively) for disseminating and implementing a practice guideline that addresses the management of croup.

#### Primary objective

To determine which of the three intervention strategies are most effective at lowering the rate of hospital days per 1,000 disease episodes. The null hypothesis is that none of the intervention strategies reduce hospital utilization rates from baseline. The alternate hypothesis is that the intervention strategies will have a graded degree of effect on hospitalization rates, with the low intensity intervention having minimal to no effect, the intermediate intensity intervention having moderate but significant effect, and the high intensity intervention having the greatest effect.

#### Secondary objective

To determine which of the three intervention strategies are most effective at increasing the use of therapies of known benefit.

#### Other objectives

To determine which intervention strategy will most effectively maintain or improve clinical outcomes and maintain or reduce the family pyschosocial burden. Clinical outcomes assessed will include both uncommon severe events, as well as average duration of clinical symptoms. The assessment of family psychosocial burden will include the number of hours of sleep missed by the child, and the stress experienced by the primary caregiver (most commonly the mother).

#### Economic analysis

To determine which of the three intervention strategies will most effectively reduce total societal costs including all health care costs, costs borne by the family, and costs stemming from the strategies for disseminating guidelines. The null hypothesis is that neither the intermediate nor the high intensity interventions will consume less resources than the low intensity intervention. The alternate hypothesis is that the intermediate intervention will consume fewer resources than either the low intensity intervention or the high intensity intervention.

## Methods

### Proposed trial design

We will conduct a cluster randomized controlled trial after completing a baseline survey. In order to provide a comprehensive answer as to whether or not our interventions are beneficial, we have obtained data from several different sources. The stages are as follows.

1. Baseline survey (currently near completion):

▪ Utilizing administrative data, we have documented Alberta health care utilization rates.

▪ We are documenting severe adverse outcomes in Alberta utilizing administrative data, and both medical examiner and records audit.

▪ Using this data, we have rank ordered Alberta hospitals based on number of disease episodes and rates of hospitalization.

▪ We have enrolled the highest ranking 24 hospitals who consented to participation.

▪ We are documenting practice patterns in these 24 selected Alberta hospitals utilizing medical records audit.

▪ Utilizing a prospective cohort questionnaire, we are documenting the duration of clinical symptoms of children with croup and the psychosocial burden on their families in the same 24 selected Alberta hospital emergency departments

2. Development of clinical guidelines that address indications for drug therapy and hospital admission/discharge criteria. The process will include:

▪ convening an guideline committee with a range of disciplines and professions;

▪ critically reviewing the published literature;

▪ drafting guidelines which meet standard criteria for developing guidelines;

▪ review of the guidelines by both parents and a wide-range of users (family physicians, nurses, and respiratory therapists);

▪ obtaining approval of the guidelines from the Canadian Pediatric Society, Canadian Association of Emergency Physicians, and the Alberta Medical Association; and

▪ publishing the guidelines in journals such as the Canadian Medical Association Journal.

3. Randomization of 24 Alberta hospitals to one of three implementation strategies.

4. A follow-up survey whose purpose is to detect any change from baseline by:

▪ documenting severe adverse outcomes and utilization rates in Alberta;

▪ documenting practice patterns in the 24 Alberta hospitals surveyed at baseline;

▪ documenting the duration of symptoms of children with croup and the psychosocial burden on their families attending these 24 hospital emergency departments.

### Proposed sample size

**Figure 1:**
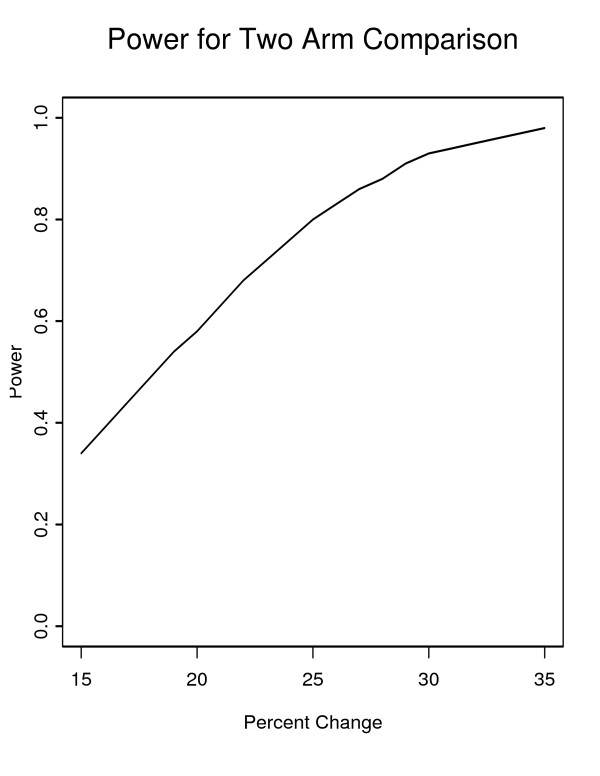
Illustration of Estimated Study Power.

The twenty-four participating hospitals were initially selected by calculating anticipated hospital specific effect sizes based on assuming that the effect of intervention would be proportional to baseline admission rates taking into account hospital-specific frequencies of disease episodes. Hospitals are to be randomized to one of three intervention arms, eight hospitals per arm, after stratification on baseline frequency of disease episodes. The power of our study depends on the anticipated change in mean hospital days as shown in Figure 1, which provides power estimates plotted against percentage decrease based on examining hospital stays over the three baseline and three post-intervention years. (Appendix A – Details of calculations) A Bonferroni correction for the two comparisons of intermediate and high intensity intervention against low intensity intervention has been included. The estimated power (overall alpha = .05) to detect a 25% change is 0.80 for each comparison.

### Recruitment

#### Recruitment of hospitals

All 24 hospitals have been approached and have consented to participate in our study.

#### Recruitment of families to complete prospective survey

As of Oct. 27^th^, 2002 we have recruited a total of 170 children into our baseline cohort and 95% have been contacted daily until resolution of symptoms. We will continue to recruit patients until April 30^th^, 2003. Given our rate of enrolment to date, we anticipate enrolling between 450 and 500 patients in the baseline cohort. The intervention and follow-up sample will be obtained over a total of 24 months, as opposed to the baseline in which we will have enrolled patients for only 13 months. Therefore, conservatively, enrolment should be ~ 900 patients in the follow-up cohort.

### Practical arrangements for allocating participants to trial groups

#### Proposed strata, rationale and method of allocation

As noted above, a total of 24 hospitals have agreed to participate in our study. We will stratify these hospitals into three levels based on the average number of croup cases diagnosed by affiliated physicians per year *(< 100 cases/year = small volume hospitals; 100 to 700 cases per year = medium volume hospitals, and > 700 cases per year = large volume hospitals)*. Non-stratified randomization of only 24 hospitals into three intervention arms could easily result in substantially more hospitals of one stratum in one intervention arm than the other arms. Therefore, given the significant differences in total number of disease episodes, number of hospitalizations, hospitalization rates, and likely other significant differences between the different sized hospitals, non-stratified randomization could significantly bias our results. A statistician not otherwise involved in our study will randomise hospitals within each of the three strata to one of the three intervention arms by computer software.

### Planned trial interventions

#### Development of croup clinical practice guidelines

##### Development committee

The Alberta Medical Association Clinical Practice Guideline Program (*Alberta Medical Association Clinical Practice Guidelines Program*) appointed DWJ as Chair, and the health care professionals representing pediatric emergency medicine (TPK, WC); pediatric pulmonary; pediatric infectious diseases; emergency medicine; rural family medicine; respiratory therapy; and nursing to a committee that was charged with developing the guidelines.

##### Criteria for developing a practice guideline

We have used both the criteria developed by Grimshaw and Shaneyfelt as the basis for formulating our guidelines.[1,2]

##### Systematic review of the literature

Utilizing the Alberta Research Centre for Child Health Evidence (ARCHE), we carried out a systematic literature review to ensure we had identified all primary published studies addressing the management of children with croup. We have completed a draft of the guidelines.

##### Remaining steps prior to completion of croup clinical practice guideline

When the guidelines are in final form, a parents' group will critically review the guidelines for clarity and content. As well, the guidelines will be reviewed by a randomly selected group of family physicians for clarity and clinical relevance. Once development of the guidelines is complete, we will seek endorsement by the Canadian Pediatric Society and the Canadian Association of Emergency Physicians. We will also submit the guidelines to the Canadian Medical Association Journal for publication.

#### The three intervention strategies

The intervention strategies to be compared will be a) mailing of printed educational materials (PEMs) (low intensity intervention); b) mailing PEMs plus a combination of interactive educational meetings, educational outreach visits, and reminders (intermediate intensity intervention); and c) mailing PEMs, interactive sessions, outreach visits, reminders plus identification of local opinion leaders (high intensity intervention.

##### Low intensity intervention

The low intensity intervention is the current strategy utilized by the *Alberta Medical Association Clinical Practice Guidelines Program *for disseminating new guidelines. This intervention arm will serve as a "control". The Croup Guidelines will be mailed to each physician registered with the *Alberta College of Physicians and Surgeons*. The Guidelines will also be available on the *Alberta Medical Association Clinical Practice Guidelines Website*.

##### Intermediate intensity intervention

The intermediate intensity intervention utilizes techniques that have consistently shown at least a moderate impact on professional behavior. We will work through existing organizational structures in order to more closely simulate how the *Alberta Medical Association Clinical Practice Guidelines Program *might disseminate future guidelines. More specifically, the *Alberta Medical Association Clinical Practice Guidelines program *and the Croup clinical practice guideline committee will work with the Universities of Calgary and Alberta Continuing Education Programs to develop an interactive educational program for physicians, nurses, and respiratory therapists. Utilizing the existing Continuous Quality Improvement Staff in each Health Region, each hospital's admitting physicians, acute care nurses and respiratory therapists will be invited to participate in the interactive educational program carried out by one of the Croup Clinical Practice Guideline Committee members. A "local champion" will also be identified. They will conduct educational outreach visits to physicians, nurses, and respiratory therapists who did not attend the interactive educational program.

##### High intensity intervention

The high intensity, intervention utilizes a multi-faceted approach described by Lomas.[3] This approach uses a variety of techniques, including interactive educational meetings, educational outreach visits, reminders, identification of local opinion leaders and establishment of a local consensus process. The physician-focused multi-faceted dissemination strategy will involve identification of physician "opinion leaders" at each hospital randomized to this arm. All physicians with admitting privileges to a hospital will be asked to complete a validated questionnaire for the purpose of identifying the "local opinion leader".[4] All "opinion leaders" will be invited to participate in a workshop on the evidence for the practice guideline's recommendations and on basic principles of behaviour change.

### Planned inclusion/exclusion criteria

#### Health care utilization using administrative databases

Alberta Health and Wellness, a branch of the Alberta provincial government, is the custodian of several data sources that are accessible for research purposes. These databases can be linked, and are considered to be of good quality with reliable personal identifiers on more than 95% of records. Through collaboration with the Project Coordinator, Health Surveillance Branch, Alberta Health & Wellness, we extracted the data specified below from the Canadian Institute for Health Information Hospital Inpatient database, Alberta Ambulatory Care Classification System, the Alberta Health Care Insurance Plan (AHCIP) Payment Database, and the AHCIP Registry Dataset.

All children 0–6 years of age and 6–16 years of age who are Albertan residents, and who have been evaluated and diagnosed by an Albertan physician to have croup (*ICD-9-CM 464.1 Acute Tracheitis, 464.2 Acute Laryngotracheitis, and 464.4 Croup, either as a primary or secondary diagnosis*) have been included in our utilization data.

##### Methodological assignment of physicians to hospital

Encrypted physician identifiers are available for each health care encounter in which a child was diagnosed to have croup. Each of the physicians appearing in our dataset were assigned to a specific hospital based on following: first, the hospital to which they admitted children with croup (62% of physicians); second, the hospital clinic or emergency department at which they diagnosed children to have croup (82% of physicians); or, third, the hospital to which a physician associate admitted or evaluated children with croup (93% of all physicians). (Physicians who shared the same billing address where assumed to be associates.)

#### Adverse outcomes

We have used administrative databases, selected medical record audit, and review of medical examiner cases to establish Alberta morbidity and mortality rates for the six-year baseline. Data gathered from these three sources will allow us to establish the incidence of rare but severe outcomes such as death, anoxic brain injury, and endotracheal intubation.

*Administrative databases *were screened for any children with a diagnosis of croup who died, were intubated (ICD-9-CM 96.04), or were also diagnosed during the same admission to have anoxic brain damage (ICD-9-CM 348.1) or cerebral edema (ICD-9-CM 348.5).

##### Retrospective record audit

We are auditing the medical records of children identified from the administrative databases who met the above criteria. The baseline review is almost complete, and we will repeat the process in follow-up. To double check that our reliance on administrative databases has not resulted in missed patients, we have also reviewed the manual admission logs for Alberta Children's Hospital and the University of Alberta Hospital intensive care units, and will contact the medical records departments at each of the regional medical centers (Medicine Hat, Lethbridge, Red Deer, Fort McMurray, Grande Prairie).

##### Medical examiner's office

We have also contacted the Alberta Medical Examiners' Offices and have arranged for review of children ≤ 16 years of age that died of "respiratory causes".

#### Practice patterns using retrospective medical record audit

Retrospective record audit will be used to establish the extent to which physicians use effective therapies for croup. The baseline review is almost completed, and will be repeated for the follow-up period. Each of the 24 targeted hospitals were asked to generate a list of medical record numbers using standard ICD9 codes as above that identify all children that were hospitalized and/or evaluated in the emergency department with croup. Medical records pertaining to all hospitalized children were (will be) reviewed. Of those children evaluated in the emergency department, a maximum of 30 patients per year were randomly selected and reviewed. For the follow-up record audit, since significant annual change is likely to occur, we will audit up to a maximum of 100 records to allow greater accuracy for our annual estimates.

#### Clinical outcomes & psychosocial burden using prospective questionnaire

The parents of all children evaluated in, or admitted to each of the 24 hospitals with a diagnosis of croup between September and May during the baseline, intervention, and follow up periods will be approached to participate in a follow up telephone survey by a staff nurse. A log of all children diagnosed to have croup will be maintained to allow the comparison of those children enrolled to those not enrolled.

#### Identification & enrolment of participating hospitals

As noted above, Alberta hospitals were rank ordered based on the number of disease episodes and rates of hospitalization for a six-year period. We then approached in this order each of the hospital administrators and clinical staff for permission to include their hospital in our study until a total of 24 hospitals consented (the staff of 11 hospitals refused to participate for reasons ranging from "anticipated closing of the hospital in the near future" to "too heavy an administrative workload to participate in a study").

### Proposed duration of treatment period

Detailed planning for the intervention strategies will take place between February and July 2003. We will initiate each of the interventions in August, 2003 and conclude them in March, 2004.

### Duration of the baseline and follow-up periods

#### Utilization and adverse outcome data obtained from the administrative datasets

We have extracted data from administrative databases from April 1^st^, 1994 to March 31^st^, 2000, providing six years of data. In summer 2006, we will again extract data from administrative databases from April 1^st^, 2000 to March 31^st^, 2006. Extraction of data across these time periods will allow us to extend the baseline utilization rates from six to nine years. Most importantly, this will ensure no significant changes occur just prior to the study interventions. Extraction of data through March 31^st^, 2006 will provide utilization data for the intervention and two follow-up years.

#### Practice pattern data obtained from medical record audit

We are just completing an audit of medical records of children diagnosed to have croup based on ICD9 coding from April 1^st^, 1994 to March 31^st^, 2000. By late 2006, we will repeat an identical audit of medical records of children diagnosed as having croup between April 1^st^, 2000 to March 31^st^, 2006. This timetable for extraction of data provides the above delineated advantages.

#### Clinical outcome & psychosocial burden data obtained from prospective questionnaire

##### Duration of enrolment

The parents of children admitted to each of the 24 hospitals with a diagnosis of croup during "croup season" (Sept 1^st ^to May 31^st^) from November 1^st^, 2001 and finishing March 31^st^, 2006, will be asked by a staff nurse to participate in a telephone survey.

##### Duration & mechanism of telephone follow up

The enrolment and consent forms are then faxed to the study coordinator (JW). She assigns the case to one of three centrally located and trained study investigators who will contact the primary caretaker within 24 hours of enrolment. The initial telephone interview takes 20 minutes. Daily telephone interviews with the primary caretaker occur until the child has been symptom-free for 24 hours.

### Proposed primary and secondary outcome measures

The primary outcome will be the rate of hospital days per 1,000 disease episodes. If differences in rate of hospital days between intervention groups are detected, we will explore to what degree they are due to a change in rates of admission versus a change in hospital lengths of stay.

The secondary outcomes will be utilization of appropriate therapies including:

▪ Proportion of patients treated in the emergency department and hospital with a corticosteroid.

▪ Proportion of patients evaluated in emergency department for at least three hours after treatment with corticosteroids before the decision to admit to hospital is made.

▪ Time to treatment with corticosteroids in both emergency department and hospital patients.

#### Other outcomes

##### Clinical outcomes

To determine which dissemination strategy will most effectively maintain or improve clinical outcomes:

▪ by reducing or maintaining the number of uncommon, severe clinical events such as intubation, respiratory and cardiac arrest, anoxic brain injury, or death.

▪ by reducing, (in a prospective cohort of children enrolled from each hospital) croup symptoms on days 1, 2, and 3 following assessment, as measured by a telephone follow-up

▪ assessment tool (Telephone Out Patient (TOP) score).

##### Psychosocial burden

To determine which dissemination strategy will most effectively maintain or reduce the psychosocial burden on parents by

▪ reducing, (in a cohort of children enrolled from each hospital), the stress experienced by the primary caregiver due to the child's illness on days 1–3 following assessment.

▪ reducing, (in a prospective cohort of children enrolled from each hospital), the number of hours of sleep missed by the child due to croup on days 1–3 following assessment.

### How will the outcome measures be measured at follow up?

#### Utilization outcomes

Previously discussed.

#### Adverse outcomes

Previously discussed in part.

##### Development of data abstraction sheet

The Principal Investigator and Study Coordinator, following an informal review of medical records, developed a customized Access relational database. This draft database was pilot tested and revised before beginning formal review.

##### Auditing process

Because these cases are significantly more complex, only the study coordinator and another trained senior pediatric emergency nurse will perform these audits. The principal investigator and the two nurses will meet regularly during the baseline and follow up audits, to discuss cases and any difficulties encountered in auditing. To establish the degree of agreement between auditors, the three will review a random selection of medical records.

##### Case identification: multiple and overlapping methods

In order to minimize the likelihood that any adverse outcomes will be missed, overlapping methods for identifying cases will be used. Specifically, we will obtain provincial medical examiner records, and hospital administrative data from the province, all the health regions, and all the hospitals in urban areas.

#### Practice pattern outcomes

##### Development of data abstraction sheet

After an informal review of medical records, a customized Access relational database was developed. This draft data sheet was piloted and revised, before beginning formal review.

##### Training & monitoring of medical record auditors

The projector coordinator has trained two research assistants to review medical records and directly enter data in a relational database (Access). The project coordinator and each of the research assistants will review a randomly selected number of records through the study to check accuracy in data abstraction.

##### Accuracy of ICD9 coding

To assess the accuracy of using ICD9 coding to identify children diagnosed with croup, we will examine whether health record analysts coded records in the same way at each of the 24 hospitals. We will determine what percentage of children were coded as croup using the standard codes met a formal definition for croup (acute onset of stridor associated with a "seal-like" barking cough). Also to explore whether some hospitals use other ICD9 codes for children with croup, we will generate a list of potential alternative codes. Using this list of potential alternative ICD9 codes, we will review a random selection of medical records using these codes.

#### Clinical & psychosocial burden outcomes

##### RMO database

We are maintaining a database of all children diagnosed as having croup whose parents refused, were missed, or are otherwise excluded. Demographics and disease characteristics of children and their families in this database will be compared to those families who are administered the survey to ascertain if the two populations are different.

##### Telephone interviewing techniques

Several procedures should enhance the reliability of telephone data collection. a) One study investigator will complete all telephone follow up for a given family. b) At the time of enrolment the primary caretaker of the child will be identified, and every effort will be made to conduct all follow-up calls with this person. c) The questions will have standardized responses. d)A standard audio recording of a child with croup will be played to the caregiver to aid them in identifying the easily recognizable and distinctive cough.

### Proposed analyses

The principal analysis will examine hospital stays arising from individual disease episodes over the six-year baseline and study period. We will apply a linear mixed model (Laird-Ware approach) incorporating random effects for hospital, year within hospital, as well as fixed effects for intervention and year. We will also pursue a more detailed explanatory analysis incorporating individual subject characteristics (age, sex) as well as random effects for admission and discharge physician. Additionally we will develop similar models for length of stay restricting to disease episodes leading to admissions, and mixed effects logistic regression model for admissions. Secondary analyses relating to steroid use and *Telephone OutPatient (TOP) *scores will follow a similar format. The examination of day 1- 3 *TOP *scores will require augmenting the basic model framework to include random subject effects and a serially correlated (within subject) residual error structure. No subgroup analyses are planned.

### Economic analysis

An economic analysis will be carried out from a societal perspective with costs classified as either payer (costs born by the province) or non-payer (costs born by individuals or the families of children with croup). The analysis will include all health care costs, costs borne by the family, and costs stemming from the strategies for implementing guidelines.

#### Potential benefits

Potential benefits will be considered to be the duration of croup symptoms and the absence of any severe adverse outcomes among children.

#### Overview of costing methods

Costing information will be gathered in sufficient detail to allow a comparison of costs and benefits. The form that the analysis will take will depend on the relative ratios of cost versus benefit. For example, if either of the two experimental strategies (non-standard) achieve similar or better clinical outcomes at decreased or equal costs as the standard dissemination strategy, the experimental interventions will be judged as cost-effective. Alternatively, if the experimental interventions yield increased benefits at increased costs, a ratio of the incremental cost per outcome gained will be calculated. And last, if the experimental interventions yield decreased benefits but are associated with increased or equal costs, the standard dissemination strategy will be judged to be the most cost-effective. (Please note we have specifically avoided using any reference to either cost-effective analysis or cost-benefit analysis. [5,6])

#### Health care costs

Payer costs resulting from the provision of health care will be estimated by first determining from the administrative database the total number of physician visits, emergency department visits, days of hospitalization, patient transports, and days in an intensive care unit (Edmonton or Calgary only). Costs per physician visit will be based on the Alberta physician fee schedule. Costs per emergency department visit, and hospital day will be based on published standards for Alberta.[7] Costs for drugs, radiographs, and laboratory tests will be based on provincial standards. For the Edmonton and Calgary hospitals included in the randomised trial and for any intensive care patients, specific hospital case costing is available thereby allowing a unique cost estimate for each patient. Air transport costs will be estimated to be $8 per air mile.

#### Implementation strategy costs

Costs of the three-implementation strategies will be classified as either as payer and non-payer based on whether or not the province assumes responsibility for the cost. They will be estimated from the costs of publication and mailing of questionnaires or learning materials, and the time spent in meetings, organising, travel, and preparing for presentations by the Alberta Medical Association support staff, Regional Quality Assurance staff, "local champions", opinion leaders, and any associates. (The hourly wage will be assumed to be $100 per hour for physician time and to be the mean provincial wage for other health care professionals and administrators.)

#### Costs to the family

The costs borne by families will be considered to be non-payer. The amount of work missed (including both wages and house work), travel time, parking and ambulance costs by each family will be assumed to equal the mean values determined by the prospective survey of children with croup. The value of the lost wages will be estimated by applying a mean wage rate to missed work time, obtained from *STATS Canada *for Alberta. The values for travel time, parking costs, and ambulance costs will be taken from published standards for Alberta.[7]

#### Calculation of societal costs

Total societal costs (the sum of payer and non-payer costs) for each hospital will be calculated for the 9 baseline years, the one-year of intervention, and the two years of follow-up. Given the year-to-year variability in the incidence of croup, the total societal costs for each year will be adjusted for disease incidence (total costs per year divided by the number of episodes of croup diagnosed that year by those physicians assigned to the hospital). An average cost per year for the baseline period will be calculated. Statistical assessment of whether or not costs differ between the three intervention groups will be calculated by the Laird-Ware model with baseline costs as the covariate. (This type of analysis will allow us to simultaneously compare costs at three time points (the intervention year and two years of follow-up), while adjusting for the baseline cost)

##### Quality of Life (QOL) measurements

We will not utilize any QOL measures in our study. None, to our knowledge, have been developed for use in children with acute, transient illnesses.

### Data safety and monitoring committee

Given that the study intervention does not pose any direct risk to patients or health care professionals, a Data Safety and Monitoring Committee is not necessary.

### Proposed methods for protecting against sources of bias

#### Masking of allocation

As noted above, a statistician not aware of our study question will allocate hospitals using a standard computer randomization software.

#### Cluster randomization

Given that our study interventions specifically target physicians and other health care workers, one threat to the validity of our study is the possibility of communication between health care workers randomized to different intervention arms. To minimize the likelihood of this possibility, we have chosen hospitals as our unit of randomization and analysis. All hospitals but three are located in different cities and towns, each with a significant distance between them. However, three of the six hospitals with large volumes are located in Edmonton, so at least two of the Edmonton hospitals will be randomized to different intervention arms.

#### Inability to completely blind health care workers to study intent

Another potential threat to the validity of our study is that – as a result of needing to approach hospital clinical staff to obtain consent for their hospital to be included in our study – hospital staff had to be informed of the intent of the study. We have tried to minimize further unnecessary contamination, however, by instructing our study staff to avoid discussing the study's main purpose with hospital staff, and speaking only about the operational details of their particular intervention.

#### Assessment of outcomes

The assessment of outcome measures will be masked as much as possible without awareness of study purpose or intervention assignment. The data analyst responsible for extracting and linking the administrative databases will not be informed as to specific purpose of the study nor to which intervention arm each hospital has been randomized. Medical record auditors will not be informed as to the specific purpose of the study nor to which intervention arm each hospital has been randomized. Parents of children with croup will not be told of the overall purpose of the study nor the intervention arm to which their hospital is randomized.

#### Analysis

The health economist (CM) will review and clean the economic data without being aware of the specific hospital or intervention (for this process, hospital and intervention identifiers will be removed). Furthermore the statistician (RB) will be provided with data segregated into the three intervention arms labelled with an encrypted hospital and intervention identifier.

## Competing interests

The author(s) declare that they have no competing interests.

## Authors' contributions

DWJ conceived of the idea for the study, devised the study design, and wrote the majority of study protocol. WC reviewed the study protocol and provided critical feedback. RB wrote the statistical analysis section of the protocol. CM wrote the health economic section of the protocol. LS provided advice on the availability of administrative data from Alberta Health & Wellness. TK reviewed the study protocol and provided critical feedback.

## Supplementary Material

Additional File 1 Appendix I – Power Calculations Click here for file
