# Supplementary material for: A cluster randomized controlled trial comparing three methods of disseminating practice guidelines for children with croup [ISRCTN73394937]
Source: Implement Sci. 2006 Apr 28;1:10. doi: 10.1186/1748-5908-1-10 (PMC1471799; doi:10.1186/1748-5908-1-10)
Supplement: Additional File 1 — Appendix I – Power Calculations [file 1748-5908-1-10-S1.pdf]

Appendix I - Power Calculations

We have not followed the classical cluster randomization sample size approach of Donner, Birkett and Buck (1981) due to the unbalanced nature of our design (varying sample sizes between clusters) and since this cluster randomized study has the major advantage of incorporating baseline data.

Our primary analysis is essentially based on comparison of before and after (within hospital) differences between treatment groups. As a consequence, some (but not all components) of between hospital variability are eliminated from consideration, ameliorating the typical variance inflation due to within hospital correlation. To allow for varying cluster sizes, we consider particular variance components contributing to experimental error as follows.

Individual observations of hospital stay duration (including 0 values for disease episodes not resulting in admissions) will be made over the three year baseline and three year post-intervention periods. Let y_{ijkl} represent the individual observation from treatment group i ($i = 1, 2$), hospital ij ($j = 1, \cdots, 8$), year k, ($k = 1, \cdots, 6$) and episode l. We assume a mixed-effects model

$$y_{ijkl} = \mu + \alpha_{ik} + \beta_k + \gamma_{ij} + \delta_{ijk} + \varepsilon_{ijkl}$$

where the α's represent fixed treatment effects, (we take $\alpha_{ik} = 0$ for the control group and for baseline years in the experimental groups and otherwise independent of year) the β's represent fixed year effects, the γ_{ij}'s represent random hospital effects, the δ_{ijk}'s account for random year to year variation within hospitals, and the ε_{ijkl} is the individual level residual error term. Letting σ^2_α, σ^2_γ, σ^2_δ and σ^2 represent corresponding variance components, consider the difference for the i, jth hospital in observed means for the baseline period minus the intervention period, d_{ij}, having variance

$$var_{ij} = 2/3 \times (\sigma^2_\delta + \sigma^2/n_{ij})$$

where n_{ij} is the yearly frequency (assumed constant here) of disease episodes attributed to the hospital. Letting $w_{ij} = 1/var_{ij}$, the optimal weighted mean difference (baseline - experimental period) in length of stay for treatment i will be

$$\hat{d}_i = \frac{\sum_{j=1}^{8} w_{ij} d_{ij}}{\sum_{i=1}^{8} w_i}$$

with variance $(\sum_{j=1}^{8} 1/var_{ij})^{-1}$. The variance for a contrast $\hat{d}_i - \hat{d}_{i'}$ (which is independent of year effects) is simply the sum of the corresponding variances, which leads to simple power calculations by formally taking n=1 with the above variance in standard two sample power formulae.

For our specific calculation, estimates of the variance components were derived from a mixed effects model fit of data from 1994 to 2000 for the 24 selected hospitals. Since the randomization will be stratified by mean number of disease episodes over this period, n_{ij}'s were estimated by taking similarly stratified means for yearly disease episode frequencies in the same period.

Reference

Donner A, Birkett N, Buck, C. (1981) Randomization By Cluster. Sample Size Requirements and Analysis, American Journal of Epidemiology, V114, pp. 906-914.
